# Reconstruction of the Distal Radius following Tumour Resection Using an Osteoarticular Allograft

**DOI:** 10.1155/2013/318767

**Published:** 2013-04-03

**Authors:** Katharina Rabitsch, Werner Maurer-Ertl, Ulrike Pirker-Frühauf, Thomas Lovse, Reinhard Windhager, Andreas Leithner

**Affiliations:** ^1^Department of Orthopaedic Surgery, Medical University of Graz, Auenbruggerplatz 5, 8036 Graz, Austria; ^2^Department of Orthopaedics, Vienna General Hospital, Medical University of Vienna, Waehringer Guertel 18-20, 1090 Vienna, Austria

## Abstract

Reconstruction of the distal radius following tumour resection is challenging and various techniques are recorded. We retrospectively analysed the outcome of five patients (one male and four females) after reconstruction of the distal radius with osteoarticular allograft, following tumour resection. Mean followup was 32 months (range, 4–121). In three of the five patients the dominant limb was affected. Mean bone resection length was 6.5 centimetres (range, 5–11.5). Two grafts developed nonunion, both successfully treated with autologous bone grafting. No infection, graft fracture, or failure occurred. Mean flexion/extension was 38/60 degrees and mean pronation/supination was 77/77 degrees. The mean Mayo wrist score was 84 and the mean DASH score was 8, both representing a good functional result. Therefore we state the notion that osteoarticular allograft reconstruction of distal radius provides good to excellent functional results.

## 1. Introduction

Although the distal radius is an untypical location for primary bone malignancies, about 10 percent of all giant cell tumour (GCT) affects this part of the skeleton. It represents the third most common location after the distal part of the femur and the proximal part of the tibia [1–4].

**Figure 1:**
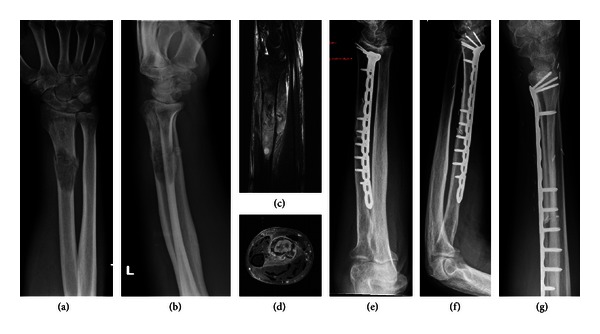
(a–g). Preoperative X-ray (a-b) and MRI (c-d) of a 64-year-old patient with osteosarcoma of the left distal radius; X-ray 22 months after replacement with allograft (e–g).

In recurrent or local aggressive cases of GCT as well as in malignant lesions, resection and subsequent reconstruction of the distal radius is indicated [2–4]. Reconstruction is challenging due to the high functional demands on the hand. Common reconstruction techniques include arthrodesis with different autografts [1, 5–9], prosthetic replacement [10–13], ulnar translocation [5, 14], arthroplasty using (vascularised [8, 15] or nonvascularised [5, 16–18]) autologous fibula graft, or osteoarticular allograft reconstructions (Figure 1) [5, 16, 17, 19–25]. 

Functional outcome as well as durability is of high importance, as affected patients are generally young with high functional demand due to their long life expectancy. Therefore, we reviewed our experience in osteoarticular allografts to assess durability, complication rates, and functional outcome of this reconstruction method.

## 2. Material and Methods

We started with searching our database for patients who received an osteoarticular allograft for reconstruction of the distal radius after tumour resection and determined age at operation, followup, resection length, complications, and revision procedures from those patients' records. General operation procedure included first, preparation and resection of the tumour including osteotomy in respect of compartmental structures. Second, preparation of the allograft and fixation of the plate on the allograft is required before; third, the plate-allograft unit is implanted and fixated to the host radius. Finally the capsule, ligaments, and eventually resected tendons are reconstructed by end to end anastomoses of the relevant anatomical structures of allograft and host.

Mayo wrist score and DASH score were used to evaluate functional outcome. The Mayo wrist score is an objective evaluation of function by comparing range of motion with the healthy side and examining grip strength and pain. A maximum score of 100 points is attainable and results are classified as excellent (91–100), good (80–90), fair (65–79), and poor (below 65). The DASH score measures general function in daily life by 30 items scored from 1 to 5, with 1 standing for no disability and 5 for maximum disability. The end score is converted to a scale from 0 to 100 where 0 implies no disability and 100 maximum disability.

Statistical analysis was done by using descriptive methods, as means and proportions, appropriate to the type of data. 

## 3. Results

**Figure 2:**
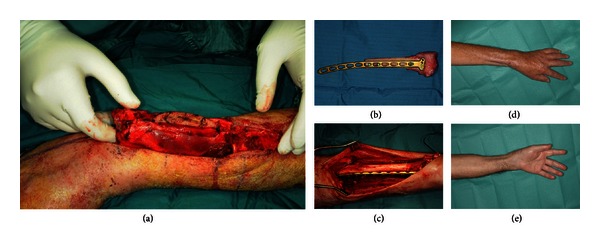
Allograft implantation in the left distal radius after resection of an osteosarcoma in a 64-year-old patient (a–c) and postoperative functional result 7 months after operation (d-e).

From 2000 to 2011 five patients, one male and four females, with a mean age of 42 years (range, 22–64) received an osteoarticular allograft reconstruction of the distal radius. Four reconstructions followed en bloc resection of a giant cell tumour and one was done after wide resection of an osteosarcoma (Figures 1 and 2).

In three of the five patients the dominant limb was affected. Mean bone resection length was 6.5 centimetres (range, 5–11.5) and mean followup was 32 months (range, 4–121). Two patients developed nonunion at the allograft-host junction, which was successfully treated with autologous bone grafting. No infections, fractures, or fixation failures occurred. 

Two patients with giant cell tumour were primarily treated with curettage and polymethylmethacrylate filling, but they experienced local recurrence. Therefore en bloc resection and subsequent reconstruction with an osteoarticular allograft was performed.

**Figure 3:**
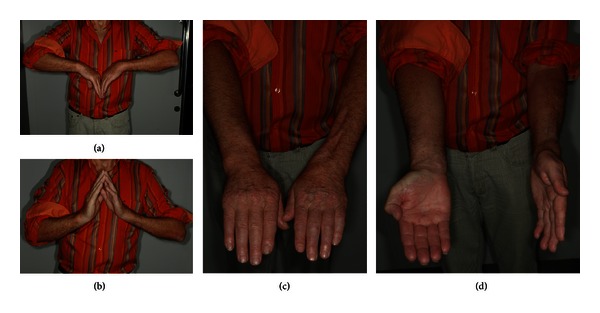
(a–d). Postoperative functional result 18 months after allograft implantation in the left distal radius after resection of an osteosarcoma in a 64-year-old patient.

The mean flexion/extension was 38/60 degrees and the mean pronation/supination was 77/77 degrees. The mean Mayo wrist score was 84 and the mean DASH score was 8, both representing a good functional result (Figure 3).

At followup none of the patients expressed pain and everyone could return to prior work.

## 4. Discussion

In a recently published clinical trial phase II, Denosumab, a RANK ligand inhibitor, achieved excellent results in treatment of GCT of the bone. In this study Denosumab significantly reduced or eliminated RANK-positive tumour giant cells and also reduced the relative content of proliferative, densely cellular tumour stromal cells, replacing them with nonproliferative, differentiated, and densely woven new bone. Denosumab continues to be studied as a potential treatment for GCTB [26]. 

At time of operation of our four patients with GCT, alternatives like Denosumab were not available. Because of tumour extension including cortical breakthrough and relatively high recurrence rates at this location, en bloc resection was the method of choice [2–4].

A skeletal defect of the distal part of the radius following tumour resection is challenging. Reconstruction as well as functional restoration is required. In patients who need a strong and stable wrist to deal with high loads, for example, manual workers, arthrodesis is the method of choice. Arthrodesis provides stability at the expense of wrist motion, which further might cause some impairment in daily life activities. Nevertheless, good postoperative results are reported in the literature with satisfying wrist function with little to no restrains [1, 5–9].

In order to preserve some wrist motion partial arthrodesis, with graft fixation only to the scapholunate portion of the carpal row, can be performed. This method provides a stable and pain-free wrist with sufficient range of motion for daily life activities [27, 28], wherefore it is recommended by Muramatsu et al. [27], especially for young patients. 

In attempt to preserve full wrist function the proximal fibula (vascularised or nonvascularised) is sometimes used for arthroplasty due to its similarity in shape and size to the distal radius. Additionally, in children the vascularised fibula provides the possibility of epiphyseal transfer and further longitudinal growth, avoiding radial club hand development. Fibula arthroplasty achieves good to excellent functional results with satisfying range of motion, but instability and degenerative change of carpofibular joint are frequently observed. Despite these complications, in most cases only minimum pain and little limitation in daily living are observed [5, 8, 15–18, 28–30]. The low level of pain is suspected to be a result of denervation of wrist joint during surgery. First, articular degeneration seemed to result from a lack of viability in nonvascularised fibula grafts. Vascularised fibula grafts, however, provide viable articular cartilage, but nevertheless, this cannot prevent joint degeneration due to the relatively incongruence of fibulocarpal articular surfaces [5, 8, 15–18, 28–30].

Osteoarticular allografts, however, offer best anatomical match with the first carpal row and avoid donor side morbidity. Further, the operation time is shorter in comparison with autografts. Good to excellent functional outcome can be achieved; nevertheless complications are still common. In contrast to allograft reconstructions at other locations, infection is rare in osteoarticular reconstructions of distal radius [5, 16, 17, 19–25]. Only Szabo et al. [20] observed one minor infection (Table 1) [17, 19–22, 24, 25], whilst the incidence of fractures and nonunion reaches up to 25% for both [17, 19–22, 24, 25]. 

In our patients all grafts were fixed with a long bridging plate and no patient suffered from an allograft fracture. Therefore, we assume that a long bridging plate presents a supportive tool to prevent fractures [16].

The relatively avascular host bed at the wrist probably favours nonunion. Two of our five patients (40%) developed nonunion, but they could be successfully treated with additional autologous bone grafting. As a possible cause we identified the use of below elbow casts for postoperative immobilisation and changed this regime to above elbow casts for the last two patients for six weeks.

The most frequent complications observed in arthroplasties with osteoarticular allografts are joint instability and articular degeneration [16, 17, 19–25]. None of the five patients in our series developed any form of instability, but recorded incidences in the literature are quite high. Cheng et al. [16] even found in all of their four study patients a translocation of the graft. In most cases only mild instability occurs without disabling patients in their working or daily life activities. Failure due to instability is rare, but instability accelerates degeneration of articular surface. Szabo et al. [20] additionally performed the Sauve-Kapandji procedure in their osteoarticular allograft reconstructions to prevent instability and found no form of joint instability at a medium followup of 8.3 years.

Degenerative changes of articular cartilage were observed in nearly all cases, but most patients reported none to only mild pain or disability and only few had to be revised due to arthritic disorders [16, 17, 19–25]. We found degenerative changes in all five patients but all were asymptomatic.

The long-term outcome and survival rates of osteoarticular allografts for distal radius still have to be studied. Our paper, as well as other studies, with short to medium followup, recorded low failure rates. Kocher et al. [22] presented the study with the longest followup of 10.9 years. They reported an average reconstruction failure of 33% after 8.1 years mean followup. This might indicate that osteoarticular allografts deteriorate with time. Long-term observations are needed to assess the real durability of this reconstruction type.

Reconstruction of the distal part of radius with prostheses as a contingent alternative to bone grafts is scarcely reported and the early attempts with them were not encouraging [10, 11]. Hatano et al. [12] reported about two reconstructions with ceramic prosthesis over ten years. Both developed radial deviation and radiolucent lines but had no clinical symptoms and achieved acceptable range of motion and function of wrist and hand. Because of radial deviation and slight instability, the author would not recommend prosthetic reconstruction in cases with more extensive soft tissue involvement to prevent subluxation and dislocation. Natarajan et al. [13] used a new designed bipolar hinge custom prosthesis in 24 patients and achieved satisfactory functional outcome and a 10-year prosthetic survival of 87.5%. Further investigations are necessary to obtain more information for considering a prosthetic reconstruction as an acceptable alternative to bone grafts.

In conclusion, although there are some problems with joint degeneration and instability, the complication rate of osteoarticular allografts is relatively low and this reconstruction method of the distal radius provides good to excellent outcome in terms of function, durability, and avoidance of donor side morbidity.

## Figures and Tables

**Table 1:** Osteoarticular allograft reconstructions at distal radius: comparison of results.

	Patients	Followup (months)	Nonunion	Infection	Fracture	Instability	Failed	Survival	Flex/ext [°]	Pron/sup [°]
Presented results	5	32 (3,7; 121)	2 (40%)	0	0	0	0	100% at 3 years	38/60	77/76,7

Scoccianti et al. [19]	17	58,9 (28; 119)	2 (11,8%)	0	2 (11,8%)	4 (23,5%)	1	94,1% at 4,9 years	56/58	80/84

Szabo et al. [20]	9	100 (39; 219)	0	1 (11%)	1 (11%)	0	0	100% at 3,5 years	26/52	80/67

Bianchi et al. [21]	12	52 (26; 145)	1 (8,3%)	0	0	7 (58,3%)	1	91,7% at 4,3 years	51/37	n.s.

Kocher et al. [22]	24	130,8 (25; 268)	0	0	6 (25%)	0	8	66% at 10,9 years	36/21	72/58

Asavamongkolkul et al. [17]*	8	52,7 (41,5; 90,9)	2 (25%)	0	1 (12,5%)	0	1	87,5% at 4,4 years	35/40	50/70

Vander Griend and Funderburk [5]*	1	36	0	0	1	1	1		n.s.	n.s.

Gitelis et al. [23]*	4	80,5 (43; 105)	0	0	0	1 (25%)	0	100% at 5 years	39/51	n.s.

Harness and Mankin [24]*	15	228	2 (13,3%)	0	n.s.	2 (13,3%)	4	73,3 at 19 years	n.s.	n.s.

van Isacker et al. [25]*	2	149.5	0	0	0	0	1	50% at 12,5 years	n.s.	n.s.

Range and percentages in brackets.

*Also other reconstruction methods than osteoarticular allografts had been used in these series.

n.s.: not specified.

## References

[1] Sheth DS, Healey JH, Sobel M, Lane JM, Marcove RC (1995). Giant cell tumor of the distal radius. *Journal of Hand Surgery*.

[2] Campanacci M, Baldini N, Boriani S, Sudanese A (1987). Giant-cell tumor of bone. *Journal of Bone and Joint Surgery A*.

[3] McDonald DJ, Sim FH, McLeod RA, Dahlin DC (1986). Giant-cell tumor of bone. *Journal of Bone and Joint Surgery A*.

[4] Goldenberg RR, Campbell CJ, Bonfiglio M (1970). Giant-cell tumor of bone. An analysis of two hundred and eighteen cases. *Journal of Bone and Joint Surgery A*.

[5] Vander Griend RA, Funderburk CH (1993). The treatment of giant-cell tumors of the distal part of the radius. *Journal of Bone and Joint Surgery A*.

[6] Pollock R, Stalley P, Lee K, Pennington D (2005). Free vascularized fibula grafts in limb-salvage surgery. *Journal of Reconstructive Microsurgery*.

[7] Murray JA, Schlafly B (1986). Giant-cell tumors in the distal end of the radius. Treatment by resection and fibular autograft interpositional arthrodesis. *Journal of Bone and Joint Surgery A*.

[8] Ono H, Yajima H, Mizumoto S, Miyauchi Y, Mii Y, Taniai S (1997). Vascularized fibular graft for reconstruction of the wrist after excision of giant cell tumor. *Plastic and Reconstructive Surgery*.

[9] Hsu RWW, Wood MB, Sim FH, Chao EYS (1997). Free vascularised fibular grafting for reconstruction after tumour resection. *Journal of Bone and Joint Surgery B*.

[10] Gold AM (1965). Use of a prosthesis for the distal portion of the radius following resection of a recurrent giant-cell tumor. *Journal of Bone and Joint Surgery*.

[11] Gold AM (1957). Use of a prosthesis for the distal portion of the radius following resection of a recurrent giant-cell tumor. *Journal of Bone and Joint Surgery A*.

[12] Hatano H, Morita T, Kobayashi H, Otsuka H (2006). A ceramic prosthesis for the treatment of tumours of the distal radius. *Journal of Bone and Joint Surgery B*.

[13] Natarajan MV, Bose JC, Viswanath J, Balasubramanian N, Sameer M (2009). Custom prosthetic replacement for distal radial tumours. *International Orthopaedics*.

[14] Seradge H (1982). Distal ulnar translocation in the treatment of giant-cell tumors of the distal end of the radius. *Journal of Bone and Joint Surgery A*.

[15] Zaretski A, Amir A, Meller I (2004). Free fibula long bone reconstruction in orthopedic oncology: a surgical algorithm for reconstructive options. *Plastic and Reconstructive Surgery*.

[16] Cheng CY, Shih HN, Hsu KY, Hsu RWW (2001). Treatment of giant cell tumor of the distal radius. *Clinical Orthopaedics and Related Research*.

[17] Asavamongkolkul A, Waikakul S, Phimolsarnti R, Kiatisevi P (2009). Functional outcome following excision of a tumour and reconstruction of the distal radius. *International Orthopaedics*.

[18] Maruthainar N, Zambakidis C, Harper G, Calder D, Cannon SR, Briggs TWR (2002). Functional outcome following excision of tumours of the distal radius and reconstruction by autologous non-vascularized osteoarticular fibula grafting. *Journal of Hand Surgery*.

[19] Scoccianti G, Campanacci DA, Beltrami G, Caldora P, Capanna R (2010). The use of osteo-articular allografts for reconstruction after resection of the distal radius for tumour. *Journal of Bone and Joint Surgery B*.

[20] Szabo RM, Anderson KA, Chen JL (2006). Functional outcome of En Bloc excision and osteoarticular allograft replacement with the Sauve-Kapandji procedure for campanacci grade 3 giant-cell tumor of the distal radius. *Journal of Hand Surgery*.

[21] Bianchi G, Donati D, Staals EL, Mercuri M (2005). Osteoarticular allograft reconstruction of the distal radius after bone tumour resection. *Journal of Hand Surgery*.

[22] Kocher MS, Gebhardt MC, Mankin HJ (1998). Reconstruction of the distal aspect of the radius with use of an osteoarticular allograft after excision of a skeletal tumor. *Journal of Bone and Joint Surgery A*.

[23] Gitelis S, Mallin BA, Piasecki P, Turner F (1993). Intralesional excision compared with en bloc resection for giant-cell tumors of bone. *Journal of Bone and Joint Surgery A*.

[24] Harness NG, Mankin HJ (2004). Giant-cell tumor of the distal forearm. *Journal of Hand Surgery*.

[25] van Isacker T, Barbier O, Traore A, Cornu O, Mazzeo F, Delloye C (2011). Forearm reconstruction with bone allograft following tumor excision: a series of 10 patients with a mean follow-up of 10 years. *Orthopaedics and Traumatology: Surgery and Research*.

[26] Branstetter DG, Nelson SD, Manivel JC (2012). Denosumab induces tumor reduction and bone formation in patients with giant-cell tumor of bone. *Clinical Cancer Research*.

[27] Muramatsu K, Ihara K, Azuma E (2005). Free vascularized fibula grafting for reconstruction of the wrist following wide tumor excision. *Microsurgery*.

[28] Minami A, Kato H, Iwasaki N (2002). Vascularized fibular graft after excision of giant-cell tumor of the distal radius: wrist arthroplasty versus partial wrist arthrodesis. *Plastic and Reconstructive Surgery*.

[29] Innocenti M, Delcroix L, Balatri A (2008). Vascularized growth plate transfer for distal radius reconstruction. *Seminars in Plastic Surgery*.

[30] Aithal VK, Bhaskaranand K (2003). Reconstruction of the distal radius by fibula following excision of giant cell tumor. *International Orthopaedics*.

